# Wearable Battery-Free Perspiration Analyzing Sites Based on Sweat Flowing on ZnO Nanoarrays

**DOI:** 10.1007/s40820-020-00441-1

**Published:** 2020-05-02

**Authors:** Wanglinhan Zhang, Hongye Guan, Tianyan Zhong, Tianming Zhao, Lili Xing, Xinyu Xue

**Affiliations:** 1 School of Physics, University of Electronic Science and Technology of China, Chengdu, 610054 People’s Republic of China; 2 College of Sciences, Northeastern University, Shenyang, 110004 People’s Republic of China

**Keywords:** Battery-free, Hydrovoltaic effect, Perspiration analyzing, Sports big data, ZnO

## Abstract

We fabricated wearable perspiration analyzing sites for actively monitoring physiological status during exercises without any batteries or other power supply. The device mainly consists of ZnO nanowire (NW) arrays and flexible polydimethylsiloxane substrate. Sweat on the skin can flow into the flow channels of the device through capillary action and flow along the channels to ZnO NWs. The sweat flowing on the NWs (with lactate oxidase modification) can output a DC electrical signal, and the outputting voltage is dependent on the lactate concentration in the sweat as the biosensing signal. ZnO NWs generate electric double layer (EDL) in sweat, which causes a potential difference between the upper and lower ends (hydrovoltaic effect). The product of the enzymatic reaction can adjust the EDL and influence the output. This device can be integrated with wireless transmitter and may have potential application in constructing sports big data. This work promotes the development of next generation of biosensors and expands the scope of self-powered physiological monitoring system.

**Electronic supplementary material:**

The online version of this article (10.1007/s40820-020-00441-1) contains supplementary material, which is available to authorized users.

## Highlights

- Wearable battery-free perspiration analyzing sites based on sweat flowing on ZnO nanoarrays was fabricated.
- Coupling of hydrovoltaic effect and enzymatic reaction were analyzed.
- The wearable wireless physiological status monitoring system has potential application in constructing sports big data.

**Electronic supplementary material:**

The online version of this article (10.1007/s40820-020-00441-1) contains supplementary material, which is available to authorized users.

## Graphical abstract

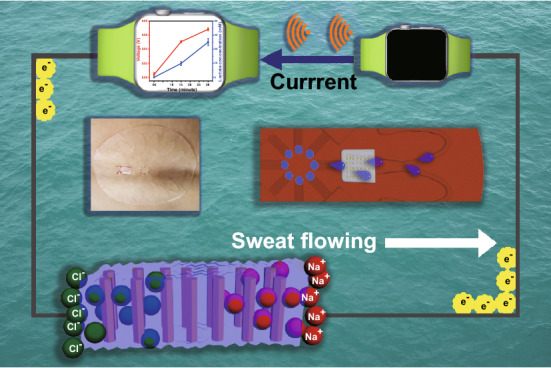

## Introduction

Wearable biosensors that interface with the human skin have received much attention because of the popularization of portable electronic consumers, such as flexible medical sensors or various smart watches/bracelets [1–6]. Recent advances in materials science, mechanics, and electronics establish the foundations for stretchable and flexible sensors that can conform to the complex, textured surface of the skin. The results enable high precision sensing through a familiar device–skin interface without irritation or discomfort [7–14]. At the same time, the rapid development of the Internet of Thing (IoT) and big data technique has also brought enormous opportunities for the advancement of traditional physiological monitoring system. Integrating passive wireless data transmission device into the system is becoming a critical component for constructing the IoT and big data. The convergence of wearable electronics, miniaturized sensor technologies, and big data techniques provides novel opportunities to improve the quality of health analysis while realizing private, accurate, real-time physiological monitoring, and data transmission [15–19].

Sweat is a vital detection factor in physiological monitoring because sweat glands cover the whole body, which provides enough convenience for vitro collection, and contains many crucial physiological indicators of physical conditions and health status [20–26]. Conventional methodologies for sweat analysis involve the collection using gauze pads taped to the skin and chemical compositional determination using benchtop instruments. Although the traditional methods are useful in laboratory and clinical contexts, these approaches cannot provide real-time information in exercises or other dynamic situations, and their accuracy is limited by loss, contamination, and degradation of samples during the multistep processes of collection, storage, transport, and analysis [6, 27, 28]. Alternative strategies exploit body-worn sweat sensors for real-time, on-skin analysis using electrochemical potentiometric and amperometric techniques. These approaches, however, may decrease his portability by including a bulky power supply, e.g., battery or capacitor [29–31]. Recently reported soft micro-fluidic systems indeed solve a lot of potential problems, but their colorimetric approaches for sensing require careful control and calibration of ambient lighting conditions, which is also difficult to achieve during exercises and dynamic state [32–34]. The perspiration analyzing sites with wireless, battery-free electronics are more promising as substitutions.

Here, we report a sort of wearable perspiration analyzing sites for actively monitoring physiological status based on sweat flowing on ZnO nanoarrays without any batteries or other power supply. As a widely used material, ZnO has attracted considerable research interest [35–42]. The device is mainly composed of ZnO nanowire (NW) arrays and flexible PDMS substrate [43]. Sweat on the skin can be inhaled into the flow channels of the device through capillary action. The hydrophobic PDMS surface is oriented along the flow channels to transfer sweat to ZnO NWs. The sweat flowing on the NWs can output a DC electrical signal. ZnO NWs generate electric double layer (EDL) in sweat, which causes a potential difference between the upper and lower ends [44]. The substance in sweat reacts with the corresponding enzyme attached to ZnO NWs, which will adjust the EDL and influence the output. In this process, the biological status transforms into the outputting DC electrical signals as sensing information. Lactic acid is an important physiological substance, and the lactate content in sweat is a common detection index in kinematics [45, 46]. Taking lactate as the monitoring target, the device can fit seamlessly with human skin and complete the data acquisition during exercises (bicycling). This device may also play an essential role in wireless construction of sports big data.

## Experimental

### Fabrication of the Battery-Free Perspiration Analyzing Sites

Sinopharm Chemical Reagent Co. Ltd. supplied all the analytical grade chemical reagents for synthesizing ZnO NW arrays.

The vertically aligned ZnO NWs were prepared by a hydrothermal method. Prior to growth, a piece of PDMS (thickness: 0.15 mm) film was cleaned with deionized water and alcohol, and dried at 60 °C. 0.5 g of Zn(NO_3_)_2_·6H_2_O was dissolved in 38 mL of deionized water. After being evenly dissolved, 2 mL of NH_3_·H_2_O was added into the solution and stirred for 10 s at room temperature, and the PDMS film attached with a clean silicon wafer (for keeping the film steady in the solution) was then immersed in. The beaker was sealed and maintained at 80 °C for 24 h. After cooling down to room temperature, the PDMS film coated with vertically aligned ZnO NWs was removed from the solution, then washed with deionized water and ethanol, and dried at 60 °C [47, 48].

A skin-like big substrate (radius 50 mm, thickness 0.5 mm) for sweat flowing was also made of PDMS. Firstly, the designed patterns and flow channels were carved on the PMMA mold using numerical control engraving. PDMS paste was then cast on the mold and sealed with a smooth flat plate. Finally, it would be kept under (80 °C) until PDMS was solidified, and the substrate was taken out.

The ZnO-grown PDMS film was cut into an area of 10 × 10 mm^2^ and attached into the corresponding position of the skin-like big PDMS substrate. Finally, ZnO NWs were modified with lactate oxidase (LOx). 0.5 mL of configured LOx aqueous solution (20u) was slowly dropped onto the surface of ZnO NWs [49]. The device was placed in a dry and ventilated place for 2–3 h. In the previous work, both ZnO and PDMS have been proven to be nontoxic/biocompatible and can work well on the human body environment [50–52].

### Characterization and Measurement

The crystal phase of ZnO NWs was characterized by X-ray diffraction (XRD, D/max 2550 V, CuKa radiation). The morphology and microstructure of the NWs and PDMS substrate were investigated by a scanning electron microscope (SEM, JEOL JSM-6700F).

The dropper aimed at ZnO NWs and dripped the test solution vertically downward. 0.4 mL × 5 times of test solution was dropped on the NWs for each measurement. It should be mentioned that for investigating the biosensing performance of the NWs, a big-size ZnO device (30 × 40 mm^2^) was firstly measured. It is a sheet with ZnO NWs made by the same method. The larger area allows the liquid flowing sufficiently to maximize the sensing gradient. The performance of standard-size device on the body-skin was then measured. The outputting voltage was measured using a low-noise preamplifier (Model SR560, Stanford Research Systems).

The standard-size device was attached on a volunteer’s chest using double-sided medical tape, and the volunteer kept bicycling during test. The outputting voltage of the device was continuously measured and recorded every 15 min. At the same time, 2 mL of sweat was collected and titrated with a commercial lactate meter for obtaining the corresponding lactate concentration.

For constructing a wireless monitoring system, a commercial piezoelectric device was used to power the wireless transmitter, and the perspiration analyzing sites can be used as a switch of the system. With or without sweat flowing through the device, the system can or cannot wirelessly transmit the information.

## Results and Discussion

**Fig. 1:**
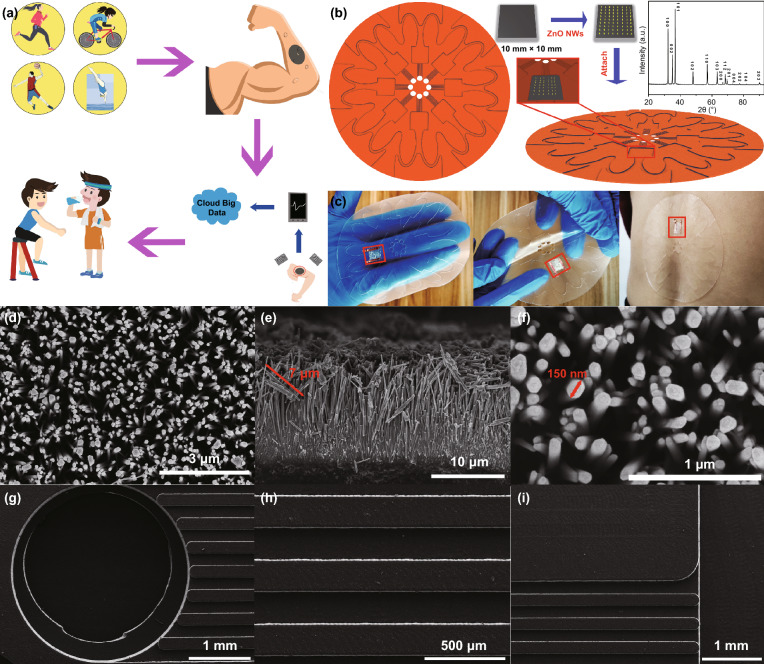
**a** Potential application of the wearable battery-free perspiration analyzing sites. **b** Fabrication process, structure display, and XRD diffraction pattern of the device. **c** Optical image of the device. **d**–**f** SEM images of ZnO NWs grown on PDMS film. **g**–**i** SEM images of the flexible skin-like PDMS substrate

Figure 1a shows the experimental design and potential application of the wearable battery-free perspiration analyzing sites. The device is attached on the skin surface of an athlete during exercises, and sweat will be inhaled into the device and flow along the channels to ZnO NWs. The outputting voltage of the device (hydrovoltaic effect) varies as the target biomolecule concentration in sweat changes. The biosensing information can be wirelessly transmitted by a wireless system. The perspiration analyzing sites on different athletes’ body can monitor and upload their physiological status for constructing sports big data.

### Device Composition

Figure 1b shows the device structure of the perspiration analyzing sites. The device is mainly composed of two parts. The sweat inlet, outlet, and the flow channels for sweat flowing are placed on the skin-like big PDMS substrate. A small PDMS film (thickness 0.15 mm) with ZnO NWs grown on is placed on the sensing position (eight positions in total) of the substrate (Fig. 1b). The XRD pattern of ZnO NWs is inserted on the right top corner, and the sharp diffraction peaks indicate good crystalline quality [53, 54]. All the diffraction peaks can be indexed to the hexagonal wurtzite structure of ZnO (JCPDS No. 36-1451). Figure 1c shows optical images of the device. It can be seen that the flexible device can adapt the skin with different deformation situations. Figure 1d–f shows SEM images of ZnO NWs grown on PDMS film. ZnO NWs are grown vertically on PDMS film and have the same growth direction with an average length of about 7 μm. From a top perspective, it can be seen that ZnO NWs grow densely and have a complete hexahedral structure with an average diameter of about 150 nm.

Figure 1g–i shows SEM images of the flexible skin-like PDMS substrate. It can be seen that the round-shape sweat inlet/outlet and the straight liquid flow channels are patterned on the substrate. The sweat inlets have a diameter of 3 mm and the width of the flow channel is 200 μm. Circle opening inlets can be defined as the sweat harvesting areas through which sweat could pass into the flow channels. The pressure drives fluid flow arising from the action of the sweat glands themselves, assisted by capillary effects in the channels. The hydrophobic surface inhibits lateral flow of sweat from the opening inlets, ensuring that fluid is inhaled into the flow channels for dominating the sweat sample. The flow channels direct the fluid to the sensing area (Movie S1). For reusability, the device is unsealed. ZnO NWs would not attach directly with the skin, which prevents material from contamination. The previous studies have verified that such size can be used for sweat flow [21].

### Sensing Performance

**Fig. 2:**
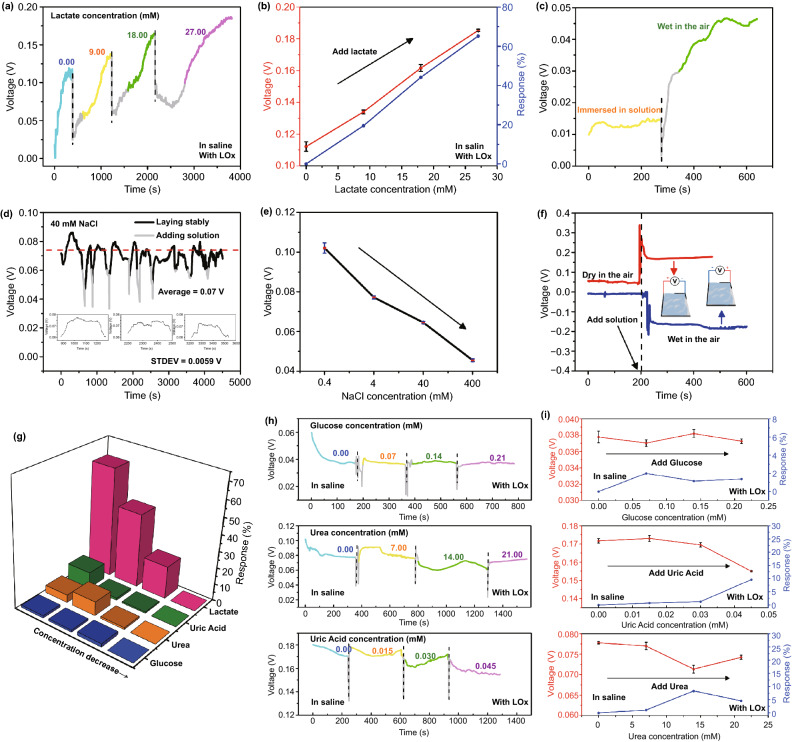
**a** Lactate biosensing performance of the device. **b** Response between the outputting voltage of the device and the lactate concentration. **c** Control experiments of primary battery’s effects. **d** Stability of the device. **e** Effect of large gradient NaCl concentration on device outputting voltage. **f** Outputting voltage of the device after switching the positive and negative terminals. **g**–**i** Selectivity of the device

Figure 2 shows the biosensing behavior of the perspiration analyzing sites. Figure 2a shows lactate biosensing performance of the device with LOx modification. When the lactate concentration in saline solution on the device surface is 0.00, 9.00, 18.00, and 27.00 mM, the outputting voltage (peak value) of the device through hydrovoltaic effect is 0.11, 0.13, 0.16, and 0.18 V, respectively. The outputting voltage increases with an increase in lactate concentration. From Fig. 2b, it can be seen that there is an approximately linear relationship between the outputting voltage of the device and the lactate concentration. The response can be simply defined as (Eq. 1) [55]:

1 $$R\% = \left| {\frac{{V_{i} - V_{0} }}{{V_{0} }}} \right| \times 100\%$$

where *V*_0_ and *V*_*i*_ are the outputting voltage of the device in saline solution with and without lactate, respectively. As the lactate concentration is 0.00, 9.00, 18.00, and 27.00 mM, the response is 0.00%, 19.46%, 44.06%, and 65.21%, respectively. The calibration curve fits Eq. 2:

2 $$y = a + bx$$

(where *R*^2^ = 0.99727, *a *= 0.00274, *b *= 0.11119; *y* is the output voltage, *x* is the concentration of lactate). The limit of detection (LOD; the signal-to-noise ratio is 3:1) can be calculated to be about 1.53 mM using the fitted linear equation [56]. As shown in Fig. 2c, in order to rule out the primary battery’s effects, the output of the device is tested in the solution and in air, respectively. The outputting voltage of the device in air is much higher than that in solution, confirming that the solution flowing is the key for generating power. The effect of primary battery can be neglected.

Figure 2d shows the stability of the device. Here, the testing environment, such as humidity, temperature, device position, solution dropping position, and solution dropping volume, keeps constant during the measurement of 5000 s. The peak values of outputting voltage fluctuate very limited. The illustrations are three enlarged view of stable voltages. Although the data fluctuate due to re-adding the solution, more statistical images show the device’s excellent stability. Figure S1a shows the distribution of an average voltage in a range of 100 s among each peak voltage. Relatively small error bars show the limited variation. Moreover, Fig. S1b shows that the linear relationship obtained by fitting each peak voltage is very close to a horizontal straight line. The device exhibits relatively high stability. Figure 2e shows that the outputting voltage of the device can be influenced by NaCl concentration. The outputting voltage decreases with an increase in NaCl concentration. When NaCl concentration changes drastically, the EDL is different. Considering that the lactate concentration in the experiment is much smaller than this range, the NaCl concentration in the testing solution needs to be constant for biosensing investigation. Figure 2f shows the outputting voltage of the device after switching the positive and negative terminals. The outputting voltage of the device is close to 0.2 V. When the positive and negative terminals are switched, the outputting voltage of the device becomes − 0.2 V.

As a lactate analyzer, the selectivity of the device is an important indicator. Several typical substances in sweat are dropped on the device with LOx modification at different concentrations, as shown in Fig. 2g–i. When the glucose concentration in saline solution is 0.00, 0.07, 0.14, and 0.21 mM, the outputting voltage of the device is 0.038, 0.037, 0.038, and 0.037 V, respectively. When the urea concentration in saline solution is 0, 7, 14, and 21 mM, the outputting voltage of the device is 0.077, 0.076, 0.071, and 0.074 V, respectively. When the uric acid concentration in saline solution is 0, 0.015, 0.030, and 0.045 mM, the outputting voltage of the device is 0.017, 0.017, 0.017, and 0.016 V, respectively. These results confirm that the device with LOx modification has relatively high selectivity for detecting lactate.

### Influence Factors

**Fig. 3:**
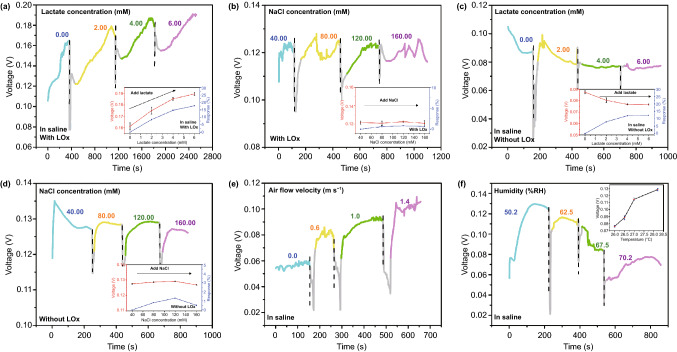
**a** Biosensing behavior of the perspiration analyzing sites against small lactate concentration. **b** Effect of small gradient NaCl Concentration on device outputting voltage. **c** Outputting voltage of the device without LOx modification against different lactate concentration. **d** Outputting voltage of the device without LOx modification against different NaCl concentrations. **e** Outputting voltage of the device with LOx modification against different air flow velocity. **f** Outputting voltage of the device with LOx modification against different humidities and temperatures

Figure 3a shows the biosensing behavior of the perspiration analyzing sites against small lactate concentration. When the lactate concentration is 0.00, 2.00, 4.00, and 6.00 mM, the outputting voltage of the device with LOx modification is 0.16, 0.17, 0.18, and 0.19 V, and the response is 0, 9%, 15%, and 18%, respectively. Figure 3b shows that the small variation of the NaCl concentration cannot significantly change the outputting voltage of the device with LOx modification. Obviously, this shows that the substance left by evaporation of sweat would not affect sensing in the short term. Since the device is designed for long-term reuse, the device needs refresh after a certain period of time. Figure 3c shows that for the device without LOx modification, the outputting voltage almost keeps unchanged with lactate concentration ranging from 0 to 6.00 mM. The response is very limited. And the small variation of the NaCl concentration also cannot significantly change the outputting voltage of the device without LOx (Fig. 3d). It is clear that enzymatic reaction plays a significant role in this biosensing process. Figure 3e shows the output of the device against different air flow. As the air flow velocity increases, the outputting voltage of the device increases, further confirming that the solution flowing is the key for generating power. Figure 3f shows that humidity temperature can influence the output of the device. As the humidity increases, the outputting voltage of the device decreases. The illustration inserted in Fig. 3f shows that the outputting voltage of the device increases when temperature rises.

### Working Mechanism

**Fig. 4:**
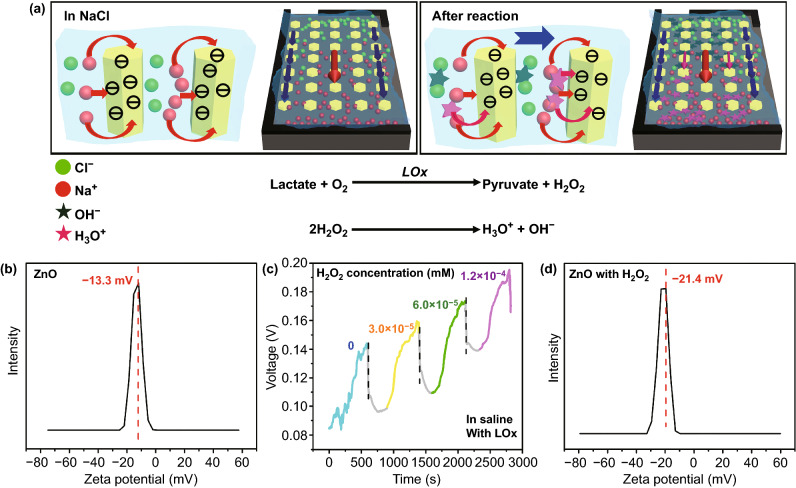
**a** Power-generating and biosensing process of the perspiration analyzing sites. **b** Zeta potential of ZnO NWs. **c** Outputting voltage of the device with LOx modification against different hydrogen peroxide concentrations. **d** Zeta potential of ZnO NWs with hydrogen peroxide

Figure 4a shows the power-generating process of the perspiration analyzing sites. The power-generating process is mainly attributed to the hydrovoltaic effect [57]. As droplets are dropped on ZnO NWs, an EDL consisting of absorbed Na^+^ and Cl^−^ ions can form at the solid–liquid interface. The accumulation of Cl ions can screen the Na^+^ ion layer adsorbed on the ZnO surface. Due to retarded migration of Cl^−^ ions, the adsorbed Na^+^ ion layer on the ZnO section just immersed in the solution will raise the local potential [58, 59]. As the solution flows, the adsorbed Na^+^ ions can accumulate at the destination of the flow, while Cl^−^ ions are hindered from accumulating at the beginning of the flow. The previous work has shown that ZnO nanofilms can generate EDL under certain conditions [57]. Meanwhile, some studies have reported that hexahedral nanostructures use flowing liquid to generate EDL [60]. Therefore, the mechanism that ZnO NWs can also generate EDL by using the hydrodynamic effect is convincing. This process results in an open-circuit voltage between the upper and lower electrodes [61, 62].

The zeta potential of ZnO NWs shown in Fig. 4b is negative, which also confirms that it can adsorb Na^+^. The channels of the device can cause the droplets to flow continuously for generating power. As the flow stops, the EDL reaches equilibrium and the output of the device decreases. It is worth noticing that every drop of solution on the surface of the device can destroy the balance of the EDL and generate power.

Figure 4a also shows the biosensing process of the perspiration analyzing sites. As the device is immersed in the saline containing lactate, the enzyme reaction between LOx and lactate can take place at the surface of ZnO NWs (Eq. 3):

3 $${\text{Lactate }} + {\text{O}}_{2} {\mathop{\longrightarrow}^{\text{LOx}}} {\text{Pyruvate }} + {\text{H}}_{2} {\text{O}}_{2}$$

Furthermore, hydrogen peroxide is oxidized as Eq. 4:

4 $${\text{H}}_{2} {\text{O}}_{2} \to {\text{O}}_{2} + 2{\text{H}}^{ + } + 2{\text{e}}^{ - }$$

Hydrogen peroxide (H^+^ and e^−^) can influence the zeta potential of ZnO NWs, and thus change the output of the device. Figure 4c experimentally confirms that hydrogen peroxide can indeed influence the output of the device. The device is immersed in saline solution containing different concentrations of hydrogen peroxide. (The concentration is roughly similar to the product of the enzymatic reaction between lactate and LOx in our biosensing measurement.) As the concentration is 0.00, 3.0 × 10^−5^, 6.0 × 10^−5^, and 1.2 × 10^−4^ mM, the outputting voltage is 0.139, 0.155, 0.171, and 0.187 V, respectively. The outputting voltage increases with an increase in hydrogen peroxide concentration. Compared to Fig. 4b, d shows that hydrogen peroxide makes a result in the absolute value of zeta potential increasing. These results further confirm that the biosensing behavior is attributed to the coupling of the enzymatic reaction and hydrovoltaic effect.

### Application

**Fig. 5:**
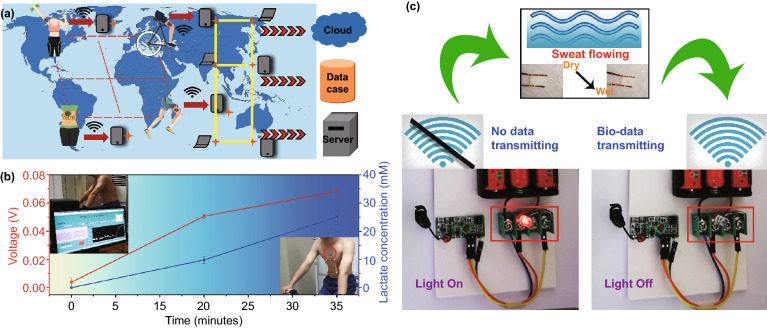
**a** Potential application scenarios of the devices integrated with wireless transmitters. **b** Experimental results of human sweat analysis. **c** Simple wireless system integrated with perspiration analyzing sites

Figure 5 shows the potential application of the wearable battery-free perspiration analyzing sites integrated with wireless transmitters. As shown in Fig. 5a, the systems attached on different athletes can real-time monitor their physiological status and wirelessly transmit the information to servers for constructing sports big data. We simply demonstrate the application in Fig. 5b. The device is attached on a volunteer’s chest during exercises (bicycling at a speed of 20.9 km h^−1^ for 30 min). At the very beginning, the volunteer cannot sweat at all, and the outputting voltage of the devices is almost zero. After 15 min, the lactate concentration in the sweat is 9.85 mM, and the outputting voltage of the device is 0.051 V. When the exercises time is 30 min, the lactate concentration in the sweat increases to 25.02 mM, and the outputting voltage of the device increases to 0.068 V. Figure 5c shows a simple wireless system integrated with perspiration analyzing sites. A commercial piezoelectric device is used to power the wireless transmitter, and the perspiration analyzing sites can be used as a switch of the system. With or without sweat flowing through the device, the system can or cannot wirelessly transmit the information. Movie S2 shows the experimental process. The solution is artificial sweat with red pigment. The wireless induction module consists of a small light bulb. When the wireless transmitter turns on (sweat flowing), the small light bulb can be shutoff.

## Conclusion

In this work, we have fabricated wearable perspiration analyzing sites for actively monitoring physiological status during exercises without any batteries. The working mechanism is based on the coupling of hydrovoltaic effect and enzymatic reaction. The sweat flowing on ZnO NWs (with lactate oxidase modification) can output a DC electrical signal, and the outputting voltage is dependent on the lactate concentration in the sweat as the biosensing signal. This device can be integrated with wireless transmitter for constructing sports big data. These present results can promote the development of self-powered physiological monitoring system.

## Electronic supplementary material

Below is the link to the electronic supplementary material.

Supplementary material 1 (PDF 112 kb)

Supplementary material 2 (MP4 1103 kb)

Supplementary material 3 (MP4 17255 kb)
