# Supplementary material for: Wearable Battery-Free Perspiration Analyzing Sites Based on Sweat Flowing on ZnO Nanoarrays
Source: Nanomicro Lett. 2020 May 2;12:105. doi: 10.1007/s40820-020-00441-1 (PMC7770781; doi:10.1007/s40820-020-00441-1)
Supplement: Supplementary file 1 — Supplementary material 1 (PDF 112 kb) [file 40820_2020_441_MOESM1_ESM.pdf]

Supporting Information for

Wearable Battery-Free Perspiration Analyzing Sites Based on Sweat Flowing on ZnO Nanoarrays

Wanglinhan Zhang[1, 2], Hongye Guan[1], Tianyan Zhong[1], Tianming Zhao[1, 2], Lili Xing[1], Xinyu Xue[1, 2, *]

[1]School of Physics, University of Electronic Science and Technology of China, Chengdu 610054, People's Republic of China

[2]College of Sciences, Northeastern University, Shenyang 110004, People's Republic of China

*Corresponding author. E-mail: xuexinyu@mail.neu.edu.cn (Xinyu Xue)

Supplementary Movie S1 Practical applications to control the flow direction of the solution

Supplementary Movie S2 A commercial piezoelectric device is used to power the wireless transmitter, and the perspiration analyzing sites can be used as a switch of the system

Supplementary Figure

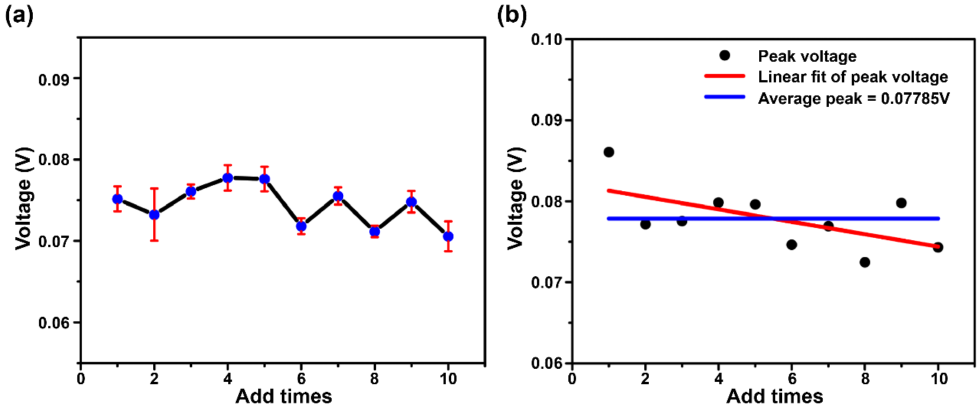

Fig. S1 a Distribution of average voltage in 100 seconds near the peak value. **b** Linear fit of peak voltage during constant experiments
